# Good participatory practice for coronavirus disease 2019 (COVID-19) research: the case of a COVID-19 prevention study

**DOI:** 10.12688/wellcomeopenres.16880.3

**Published:** 2022-12-01

**Authors:** Carlo Perrone, William Schilling, James J. Callery, Elizabeth A. Ashley, Mary Chambers, Hannah Chase, Piyush Dahal, Nipaphan Kanthawang, Supalert Nedsuwan, Borimas Hanboonkunupakarn, Daranee Intralawan, Abhilasha Karkey, Mayfong Mayxay, Vimalay Souvong, Hien Tran Minh, Summita Udas Shakya, Sanjib Kumar Sharma, Surendra Uranw, Souphaphone Vannachione, Charles Woodrow, Nicholas J. White, Phaik Yeong Cheah

**Affiliations:** 1 John Radcliffe Hospital, Oxford, UK; 2 The Ethox Centre, University of Exford, Oxford, UK; 3 Mahidol Oxford Tropical Medicine Research Unit (MORU), Bangkok, Thailand; 4 Centre for Tropical Medicine and Global Health, Nuffield Department of Medicine, University of Oxford, Oxford, UK; 5 Lao-Oxford-Mahosot Hospital-Wellcome Trust Research Unit (LOMWRU), Vientiane, Lao People's Democratic Republic; 6 Oxford University Clinical Research Unit (OUCRU), Ho Chi Minh city, Vietnam; 7 Medical Sciences Divisional Office, University of Oxford, Oxford, UK; 8 KHDC Program, B.P. Koirala Institute of Health Sciences, Dharan, Nepal; 9 Primary Care Department, Chiangrai Prachanukroh Hospital, Chiang Rai, Thailand; 10 Faculty of Tropical Medicine, Mahidol University, Bangkok, Thailand; 11 Oxford University Clinical Research Unit (OUCRU-Nepal), Patan Hospital,, Kathmandu, Nepal; 12 University of Health Sciences in Lao P.D.R., Vientiane, Lao People's Democratic Republic; 13 Department of Internal Medicine, B.P. Koirala Institute of Health Sciences, Dharan, Nepal

**Keywords:** Good participatory practice, public engagement, Coronavirus Disease 2019 (COVID-19), prevention, clinical trials, bioethics

## Abstract

**Background: **The COPCOV study (chloroquine/ hydroxychloroquine prevention of coronavirus disease), which started recruitment in April 2020, is a multi-country double-blind, randomised, placebo-controlled trial which is being conducted in healthcare facilities involved in COVID-19 case management. Participants are staff employed in facilities managing people with proven or suspected COVID-19. As part of the study, we conducted a series of engagement sessions. The aims were to assess the feasibility of the study, to identify context-specific ethical issues, to understand possible concerns, to fine tune research procedures and to refine the COPCOV information materials.

**Methods:** The COPCOV study was approved by relevant institutional review boards. The sessions described in this paper were part of the study. We conducted a series of engagement sessions, each involving a short presentation of the study, a section where attendees were asked to express their willingness to participate in such a study, which information they would need to change their view and an open Q&A section. Answers were transcribed and coded into themes by two independent investigators. Themes were derived from the data. They complemented other site-specific engagement, communication, and public relation activities such as press releases and websites.

**Results and conclusions:** From 16 ^th^ March 2020 to 20 ^th^ January 2021, 13 engagement sessions were conducted in Thailand, Laos, Vietnam, Nepal and the UK involving 222 attendees in total. Issues raised revolved around the social value and study rationale; safety of trial medications and risk-benefit balance; study design and commitments. These sessions helped us identify  concerns people had, which helped us refine information materials as well as complement site feasibility assessments. Our experience strongly supports the use of participatory practices prior to conducting clinical trials.

## Introduction

### Good participatory practice and COVID-19 trials

In the context of a novel and rapidly spreading outbreak such as COVID-19, it is essential for clinical research to be planned, approved and carried out as quickly and as effectively as possible. Indeed, since the start of the outbreak stakeholders have aimed to mobilise vast resources and streamline regulatory practices, stimulating a response of unprecedented proportions in the scientific community. At the time of writing, over 300 new papers on COVID-19 are being published daily with a total of over 150,000 publications ^1^. However, in this race against time to find therapeutics for COVID-19, it is essential that all key ethical issues intrinsic to clinical research are not overlooked and that the interests of potential participants and other stakeholders are safeguarded. In addition to the scrutiny by ethics committees and regulatory bodies, good participatory practice (GPP), or variations of it, stakeholder and community engagement, have been highlighted in various guidance documents and literature as important tools ^2– 6^.

For example, GPP for emerging pathogens (GPP-EP) ^6^, inspired by the widely-endorsed GPP guidelines for biomedical HIV prevention trials ^4, 5^, provides a set of guidelines on how to effectively engage stakeholders in the design and conduct of prevention and treatment trials for emerging and re- emerging pathogens. The 2016 Council for International Organizations of Medical Sciences (CIOMS) guidelines states that “Researchers, sponsors, health authorities and relevant institutions should engage potential participants and communities in a meaningful participatory process that involves them in an early and sustained manner in the design, development, implementation, design of the informed consent process and monitoring of research, and in the dissemination of its results.” ^7^.

Engagement around research studies occurs along a spectrum: from reaching out and informing, to consulting, involving, collaborating and formal partnerships. Engagement can be conducted in a variety of ways, such as consultation with community advisory boards ^8, 9^, participatory and community drama ^10– 14^, participatory visual methods ^15^, radio chat shows, holding village meetings and local events.

There are many challenges associated with GPP. The aims of GPP and how to evaluate GPP are not always clear, and the health research and community contexts in which they are conducted are wide ranging ^16– 18^. The goals include a combination of instrumental goals and ethical goals such as respecting stakeholders, building relationships, determining appropriate benefits, minimising risks, supporting consent processes, understanding vulnerabilities and researcher obligations, gaining approvals and building legitimacy for research ^19, 20^. In practice however, engagement initiatives in health research often have more than one goal, and the distinction between instrumental and ethical goals are sometimes unclear ^20^.

The potential unintended negative impacts should not be overlooked. These include the potential for engagement to be tokenistic, further marginalizing communities who are difficult to reach, and at risk of physical and mental exhaustion from the requirements of engagement that may not always outweigh the expected benefits ^19, 21^.

Implementing GPP requires effort, funding, resources, and expertise, but when implemented appropriately it can enhance the impact of trials such the PREVAIL (Partnership for Research in Ebola Virus in Liberia) and the recently reported RECOVERY (Randomised Evaluation of COVID-19 Therapy) trials ^3, 22, 23^. Engagement has also been shown to build trust between the community and members of the study team ^24^, strengthen the informed consent process ^25^, and help with recruitment into studies ^26^. These reasons as well as the collective experience of engaging with communities of the authors are the inspiration of the work reported here.

### The COPCOV study

COPCOV (chloroquine/ hydroxychloroquine prevention of coronavirus disease), is a randomised, placebo-controlled pre-exposure prophylaxis study to determine whether chloroquine or hydroxychloroquine prevents COVID-19 ^27^. It is being conducted in healthcare institutions around the world where proven or suspected COVID-19 cases are found. Participants are being recruited among unvaccinated healthcare workers and other persons at risk of contracting COVID-19. At the time of the engagement sessions, vaccines were not widely available yet. Participants are randomised to receive either the intervention, consisting of chloroquine or hydroxychloroquine (depending on local regulations and availability) or placebo.

All participants continue to take the usual precautions for protection against the virus. Participants take the study drugs each day for a period of three months and are followed closely to see how well the drug is tolerated, whether they contract the infection, and if they do, whether they develop mild or more severe COVID-19. If a participant develops COVID-19, they will be treated according to local treatment guidelines.

Due to the use of hydroxychloroquine/chloroquine for rheumatological conditions and for malaria, both as prophylaxis and in mass drug administration, there are large amounts of data supporting the safety of long-term administration of these drugs ^28^. However, no conclusive evidence of benefit in COVID-19 pre-exposure prophylaxis has so far been produced. Similarly, no other chemoprophylactic agents have been proven to be effective. The rationale behind usage is based on *in vitro* antiviral activity of chloroquine and hydroxychloroquine on severe acute respiratory syndrome coronaviruses (SARS-CoV1 and SARS-CoV2) *in-vitro*. It remains unclear if this will translate into clinical benefit ^29^, and we may yet still not find out ^30^.

The first COPCOV participant was enrolled in Thailand on 29 ^th^ April 2020. Within weeks of the study start, COPCOV recruitment was paused hours after an article by Mehra *et al*. was published (on May 22 ^nd^ 2020) describing increased mortality in patients receiving chloroquine/ hydroxychloroquine ^31^. The article was retracted on June 4 ^th^ 2020 because of concerns about the veracity of the dataset but by that time it had already had profound repercussions for the COPCOV trial and other trials using hydroxychloroquine. Other milestones pertinent to the COPCOV study were the announcement that chloroquine/hydroxychloroquine is not effective for the treatment of active COVID-19 by the World Health Organisation (WHO) on July 4 ^th^ 2020 and the positive results of the first studies of vaccines against COVID-19 in late 2020 ^32– 34^.

In the present paper we describe a set of engagement activities that took place as part of the development and implementation of the COPCOV study from March 2020 to January 2021 and share some insights and reflections from our experience. We also describe how these engagement activities impact the COPCOV trial and communication approaches. The specific objectives of these engagement activities were to assess feasibility of the study at the respective sites, to identify potential context-specific ethical issues, and to understand concerns participants might have. The engagement sessions were also aimed to help fine tune our research procedures and refine COPCOV information materials. These activities complemented other site-specific engagement activities, consultations with community advisory boards and public advisory groups, as well as communication and public relation activities such as press releases and websites.

## Methods

### Design

From 16 ^th^ March 2020 to 20 ^th^ January 2021, 13 engagement sessions were conducted in Thailand (4 sessions), Laos (1 session), Vietnam (1 session), Nepal (5 sessions) and the UK (1 session) with potential COPCOV participants. The details of each session are summarized in Table 1. The locations of the engagement events were purposively selected based on geographical spread, availability of facilitators and other feasibility factors including COVID-19 restrictions. The number of sessions per location varied according to the judgement of local facilitators who took into consideration the need to include a wide range of potential participants and to provide an environment conducive for participants to express their views.

**Table 1.** Overview of sessions.

Site and session number	Attendees, number	Facilitators and relationship to attendees	Attendees, background
1-The Mahidol-Oxford Tropical Medicine Research Unit (MORU), Bangkok, Thailand	70	2 physicians, 1 public engagement staff - -Co-working relationship	Researchers and academic staff, physicians, administrative staff (breakdown unknown)
2-Mahidol University, Faculty of Tropical Medicine (FTM), Bangkok, Thailand	5	1 physician Co-working relationship	Nurses (4) Admin staff (1)
3-MORU Health Research and Interest Group (HREIG) ^35^	9	3 physicians 1 public engagement staff No relationship	General public, not employed in the healthcare sector (9)
4-Chiangrai Clinical Research Unit (CCRU) Chiangrai, Thailand	4	1 research nurse, 1 physician Co-working relationship	Research nurses (2) Lab technicians (2)
5-The Horton Hospital, Oxford University Hospitals (OUH) NHS Foundation Trust, Banbury, United Kingdom	15	1 physician, 1 medical student Co-working relationship	Nurses (5) Physicians (5) Clinical support workers/Nursing assistants (5)
6-Mahosot Hospital, Vientiane, Laos	25	4 physicians 1 co-working relationship 3 no relationship	Physicians (25)
7-Chiangrai Prachanukroh Hospital, Chiangrai, Thailand	25	1 research nurse, 1 physician No relationship	Nurses (3) Physicians (17) Public officers (2) Pharmacist (1) Other (2)
8-Hospital for Tropical Diseases, Ho Chi Minh City, Vietnam	11	1 public engagement coordinator 2 physicians No relationship	Nurses (7) Physicians (3) Medical assistant (1)
9-13 B.P. Koirala Institute of Health Sciences Dharan, Nepal (5 sessions in total)	58 (total)	1-3 Physicians (depending on session) No relationship	Unreported (12) Nurses or nurse aids (35) Data managers (1) Medical officers/physicians (10)

### Approvals and ethical considerations

The protocol of the COPCOV study listed public engagement activities as part of the study ^27^. The protocol was approved by the Oxford Tropical Research (OxTREC, reference number 25–20) and local ethics committees. In accordance with the approved protocol, attendees were informed that the findings of these engagement sessions would be included into reports and, in some of the sessions, recorded for note taking and auditing purposes. Participation in these sessions was voluntary. Verbal consent was provided. In addition, active contribution to the sessions through statements, questions or comments was viewed as implicit consent to use and quote such information. No personal information with the exception of profession was collected. All these sessions were conducted with the approval of relevant authorities and held in compliance with all relevant local site-specific guidances that govern the conduct of such engagement activities. 

### Procedure

The sessions were conducted face-to-face in the place of work of participants (e.g. meeting rooms in hospitals and affiliated universities), with the exception of one session that was conducted online due to COVID-19 restrictions. The following authors were facilitators of one or more sessions: PYC (MSc, PhD), CP (MD), NK (BSc), SU (MPH, PhD), HTM (BSc), MM (MD), EAA (MBBS, PhD), SKS (MD, DM), BH (MD, PhD), CJW (MBBS, PhD). All facilitators were trained by the head of engagement of the COPCOV study (PYC). All those presenting the study and answering questions had previous research or clinical experience and biomedical training and were familiar with the study protocol and COVID-19. At the beginning of each session facilitators introduced themselves, their professional background and explained their roles with respect to the COPCOV study.

Sessions were facilitated by researchers working at the study sites, in some cases they had official duties at the institutions. See Table 1 for a breakdown of locations and number of participants and dates of the engagement sessions as well as the relationship between attendees and facilitators. The number of participants in each session was based on feasibility factors, including COVID-related restrictions. The duration of each session and the number of sessions per site also took into account when data saturation was reached. Facilitators advertised the sessions directly or liaised with representatives or collaborators of the partner institutions who in turn invited healthcare workers and researchers to attend each session. Sessions were aimed at one or more healthcare worker groups and researchers but other interested individuals were free to join, there were therefore no strict inclusion or exclusion criteria.

The COPCOV engagement team conducted engagement sessions in Bangkok and encouraged study staff at potential study sites to do the same, proposing a general format that could be adapted to local requirements: Participants of the sessions were briefed on the COPCOV study design and procedures (10–20 minutes) and asked two predefined questions in the local language: 1) “Would you take part in such a study and why?” and 2) “What additional information would strengthen or make you change your decision?”. These questions were posed primarily to stimulate discussions rather than to quantify the proportion of those who would versus those who would not take part. Participants were given up to five minutes to write their answers (or questions) down individually. This step was important to encourage all participants to voice their opinions and to provide anonymity. This was followed by a question-and-answer session and open discussions. In some sessions, additional questions were posed to attendees to facilitate discussions. All sessions were conducted by facilitators who were familiar with COVID-19, the COPCOV study, and spoke the local language.

### Data management

Participant responses were either written on sticky notes or spoken out directly. Sticky notes were then collected by the facilitators. Depending on the available time, a variable proportion of issues were discussed by the participants together with facilitators and involved researchers. Detailed notes were taken during the discussion session. Session reports were compiled by facilitators and moderators at each site and included the number of participants divided by profession, the duration, the transcription of the statements on sticky notes or spoken out as well as questions asked and the answers given. No participant identifying or demographic data (with the exception of profession) were collected.

All responses and questions were compiled into a Microsoft Excel (Microsoft Office professional 2019) spreadsheet and then coded by two members of the team (CP and PYC). Discrepancies were discussed until consensus was reached. We used the thematic analysis approach. The analysis was inductive in nature.

## Results

We conducted a total of 13 sessions involving 222 participants. No participant characteristics other than their profession were recorded. As participation was voluntary, there was a risk for selection bias towards participants interested in the project. Each session lasted between 45 and 90 minutes. Many participants were keen to join the study, while others had concerns ^36^. The responses to the pre-defined questions and open discussions were grouped into three broad themes. Each of the themes are discussed in turn below: social value and study rationale, safety and risk benefit balance, study design and commitments.

### Social value and study rationale

During each session, there was considerable interest in the rationale of the study. While many participants saw the need for the study, others questioned the rationale, both in terms of choice of trial medications and the need for a prevention trial.

A participant from Vientiane, Laos said he would participate in the trial “to prove if chloroquine can be used or not for the prevention of COVID-19” (April 2020). The need to protect oneself was also cited as a reason to participate in the study, e.g., “increase chance to protect myself and others” (Bangkok, TH, Nov 2020) and “may reduce severity of COVID in me” (Banbury, UK, Mar 2020), was a common statement.

A participant from Dharan, Nepal, who also saw the need for the study said “there is no other options so far, such as no vaccine” (Nov 2020). This was despite the fact that vaccines were already close to approval and rollout at the time in the UK and USA. This participant recognised that it would take several months to years before a vaccine could be available to the wider community in Nepal.

Many statements and questions concerning evidence and rationale referred to the limited amount of evidence or perceived lack of plausible efficacy of chloroquine/ hydroxychloroquine on SARS-CoV-2 (severe acute respiratory syndrome coronavirus 2): “I want to see case study that has been experimented on all genders, all ages and experiment with patients with underlying diseases” (Chiangrai, TH, Apr 2020) or “[…] we do not have any confirmation if chloroquine can be used for the prevention [of COVID-19]” (Vientiane, LA, Apr 2020). 

Other concerns were related to the media e.g., “Media influence that chloroquine is not effective for COVID prevention” (Dharan, NP, Nov 2020).

In addition to questions on the rationale of the trial medications, participants also questioned the need of a prevention trial e.g., when asked “what information would change your decision?”, a participant who said he was not interested in the study answered, “if it was aimed at treatment rather than prophylaxis” (Banbury, UK, Mar 2020). 

Where disease incidence was low and where personal protective equipment (PPE) was widely available, many felt that a prevention trial was not needed e.g., ”[…] would these people not be taking extra precautions like PPE? Will there be high enough risk of infection to determine the difference between the group[s]?” (Bangkok, TH, Mar 2020) and “I can use other methods to prevent myself from COVID-19” (Vientiane, LA, April 2020).

Despite emphasising that the COPCOV study is a prevention trial, some participants also talked about treatment e.g. “I want to know the treatment of COVID-19” (Vientiane, LA, April 2020).

### Concerns about safety and risk-benefit balance

An issue that was widely raised was that of safety of the trial medications. In some instances, concerns were expressed in broad terms: “need more info[rmation] on side effects and risks of the drugs” (Banbury, UK, Mar 2020) or “afraid of side effects” (Bangkok, TH, Mar 2020) In other cases, participants referred to specific side effects of chloroquine and hydroxychloroquine such as cardiovascular, ocular, hepatic or renal adverse effects. This worry was exacerbated after the article by Mehra *et al.* was published in the Lancet on the 22nd of May 2020 (the article was later retracted). In Vietnam, where the engagement session was held a few weeks after the publication of the Mehra *et al*. paper, none of the participants in the workshop were interested to join the study, “Some paper showed that chloroquine is not effective for COVID-19 patients, this drug can even cause some side effects, especially on cardiovascular system”, (Ho Chi Minh City, VN, June 2020).

In some cases, participants viewed that the risk of the side effects outweighed the risk of getting infected. Consider this quote, “I worry about side effects, and I think the chance of getting infection is low” (Chiangrai, TH, April 2020).

Participants were also interested in the management of adverse events and of complications or of COVID-19 *:* “how can we minimise the danger of side effects?” (Bangkok, TH, May 2020) or “management approach if […] side effect[s] occur.” (Chiangrai, TH, Apr 2020).

A handful of participants perceived that participating in the study would increase the risk of contracting or transmitting COVID-19: “it would put my family members at risk” (Bangkok, TH, March 2020) or “having children in case anything happens to me” (Banbury, UK, Apr 2020). Upon further probing, we realised that these participants thought that the trial would challenge participants by infecting them with the virus.

One participant expressed this concern in more detail, worrying that follow-up visits would increase contact with potentially infected people: “there will be a group of [other] people coming, it might be [at a] follow-up [visit] […] It probably increases the risk to contact […] or not? [...]” (Chiangrai, TH, Mar 2020).

### Study design and commitments

Participants were also interested in the procedural details of the study such as “when does the study start?” (Vientiane, LA, Apr 2020), “How many visits/ attendances required, Any exclusion criteria?” Banbury, UK, Mar 2020). Critical views on certain design choices were also brought up: “3 months duration sounds too long” (Bangkok, TH, Mar 2020) or “There could be many confounding factors between control and placebo” (Chiangrai, TH, Apr 2020).

These questions reflected both a desire to find out more about the study and understand the rationale of the procedures as well as concerns over research-related burdens and commitments. Examples of worries specific to the latter *:* “I worry that I would be given a placebo which has no effect” (Chiangrai, TH, Mar 2020), and “I want to join but because there are many procedures to do, such as checking temperature every day, reporting symptoms every day, and must take medicine every day, therefore, I may not have time to participate” (Chiangrai, TH, Mar 2020).

## Discussion

### Implications on the COPCOV trial

In this paper, we report our findings from twelve engagement sessions organised to follow a specific format as described above in geographically diverse locations between March 2020 and January 2021. The information gathered from these initiatives complemented other engagement strategies conducted such as discussions with site-specific investigators and local ethics committees as well as consultations with local community advisory boards and public advisory groups. The issues considered important, as derived from statements on desire to participate or not to COPCOV and open questions by attendees, were highly variable depending on location and timing. The information gathered provided important insights to improve the ethical and operational aspects of the COPCOV study.

By conducting engagement sessions of this type, investigators confirmed that the safety of the trial medications were of key importance to potential participants. Many such worries could have been prompted by media reports particularly after the publication of a likely fraudulent paper stating that hydroxychloroquine was found to increase mortality in COVID-19 patients ^31^.

Our engagement sessions also identified some delicate themes. One worrying misunderstanding was that participants thought they might be at higher risk of contracting COVID-19 by joining the study because they thought that the trial would challenge participants by infecting them with the virus. Another example was the confusion between the concepts of treatment and prevention, so some participants did not understand the rationale for conducting a prevention trial like COPCOV after preliminary data from the RECOVERY trial showed that hydroxychloroquine was ineffective in the treatment of patients hospitalised with severe COVID-19 ^23^. COPCOV studies chloroquine and hydroxychloroquine for the prevention of COVID-19 before an individual is exposed or infected, which is substantially different from the treatment of patients that already have COVID-19 or treatment of individuals who have already been exposed to an infected contact. Subsequently, it has been recognized that there are distinct phases of disease with distinct pathophysiology and treatments which are effective. Lack of efficacy in late treatment did not preclude efficacy earlier, and the answer to the question remained important for those without access to vaccines, or if vaccines became less effective ^30^.

These sessions were helpful in facilitating the refinement of COPCOV study key messages on the COPCOV trial website and participant information materials.

For example, to address the worry about being at more risk of getting COVID-19, we updated the participant information sheet and FAQ section of the COPCOV website to include “Am I at a greater risk of developing COVID-19 if I take part in the study?”

In addition to updating print information, the COPCOV team collaborated with an animation team to produce a video to summarise the safety information of chloroquine and hydroxychloroquine and to explain the difference between prevention and treatment in the context of COVID-19. In the animation, we used fire as an analogy to infections, “It is easier to prevent a fire, or put the fire out early, rather than late, when the damage is done. The same is true with infections.”

We also conducted many webinars and set up information booths at participating sites and at medical conferences (virtual) were also organised such as the American Society of Tropical Medicine and Hygiene (ASTMH).

These engagement sessions complemented the routine good clinical practice and quality-focused site assessments at potential study sites. The desire to participate in the COPCOV study varied, so the sessions were helpful in evaluating potential study sites and contributed to the decision of excluding two sites (Chiangrai, TH and Ho-Chi-Minh, VN), saving considerable time and resources. In the context of COVID-19, even if reliable data on the status of the epidemic is widely available, it is difficult to anticipate how potential subjects feel about its spread, its handling by local authorities and the need for preventive medication. Taking the examples of Vientiane (Laos) and Chiang Rai (Thailand) which are 600km apart, both sessions were conducted roughly at the same time and both cities had relatively low local SARS-CoV-2 transmission, yet 78% of participants in Vientiane compared to none in Chiang Rai said they would like to participate in the study. It is possible that views at the national, local, or even at the hospital level might have influenced these striking differences. In Vietnam, at the time of the engagement session (June 2020), none of the participants were interested in enrolling in COPCOV. In Nepal we conducted five sessions over 4 months where we noted a change in public opinion about the study which triggered the re-instigation of the study in 2021.

### Strengths, limitations and lessons learnt

As for strength, these engagement sessions were organised prior to the study being considered or launched at the respective sites. This gave us an opportunity to refine our study materials, create new information materials, and in some instances (e.g. Ho Chi Minh City and Vientiane) contributed to the decision of whether the trial would be feasible at the respective sites. This was possible because of existing working relationships between the sites and the COPCOV core team. Additionally, the primary target population of COPCOV consisted of healthcare workers, it was therefore possible to engage participants relatively easily and it was not necessary to explain research concepts in detail, as most participants were familiar with them already, which is unlikely to be the case for most clinical trials.

As for limitations, in the case of a large multi-centre trial in a rapidly evolving pandemic, with a relatively fixed design such as COPCOV, the potential for any single engagement activity in shaping significant aspects of the trial such as study design and choice of control group is limited. These high-level discussions were conducted with funders, site investigators, ethics committees and key stakeholders in the relevant countries at the funding application stage, who had provided relevant input. As engagement activities may influence any phase of research, including the choice of research topics, study design and recruitment strategies, their impacts are likely to be strongest when started early in the course of a study ^2, 6^. This is not always possible, especially in a pandemic. Due to the rapidly changing landscape of the pandemic as well as the perception of the study risk-benefit ratio, engagement with local communities and potential study participants at the earliest opportunity as illustrated by our experience was nevertheless a worthwhile exercise.

Due to the timing of the engagement sessions, there was a risk that participants felt their concerns were unanswered. The limitations of such engagement must be made clear to participants but they should also not be an excuse for not engaging with communities. It is the responsibility of the researchers to define which aspects can be adapted, explain this to engagement session participants and make sure there are sufficient resources and will to make the necessary changes after receiving feedback ^37^.

Our engagement sessions were designed to obtain quick feedback from potential participants of the COPCOV study and not for in-depth discussions on the major aspects of the trial such as study design and choice of control groups. Rather, our engagement sessions were designed to help us identify the priorities, concerns and attitudes of potential participants, refine information materials, identify site specific issues not otherwise identified by site investigators and ethics committees. These sessions did not replace, but complemented and informed in-depth informed consent sessions with potential COPCOV participants. Detailed discussions and training were also held with site staff during site initiation meetings, where detailed discussions on study procedures took place.

The majority of participants who attended the engagement sessions were healthcare workers or those who work in healthcare facilities. We chose to engage with this group because they are potential participants for the COPCOV study. As most were educated in medicine or allied health subjects, their concerns about testing drugs were based on good prior knowledge.

We did not conduct these engagement activities at all participating sites. This was primarily due to the feasibility and practical reasons such as availability of experienced engagement staff at the respective sites. Should all sites have had engagement staff, as recommended by GPP guidelines, the positive impact of the engagement activities would have been even greater ^5, 6^. However, other forms of engagement took place at each site, following local guidelines in Kilifi, the local team engaged with the KEMRI Community Representatives ^9^. The specific findings of engagement other than that described in our methods are not reported here.

The CIOMS 2016 guidelines recommends that ‘researchers and ethics committees should be cognizant of the point at which the process of community engagement becomes a stage of formative research that itself requires ethics review. Examples of community engagement processes that may require ethics review include systematic data collection that can be generalized and disseminated in forums outside of the community in which they were implemented, as well as any data generation that could create social risks for participants’ ^7^. Our engagement activities were not designed as a research study in their own right. We did not seek specific ethics approval but they were approved as part of the COPCOV study. We believed what we learned would be useful to the research community and we therefore described our experience in this paper. Participants were informed that the sessions would be recorded but we did not obtained written informed consent. It was not a requirement set out by our ethics committees. Participants were free to leave the sessions if they did not agree to being recorded. Each participating site performed the engagement activities as they saw most fit for their site, taking local cultural and logistic factors into account, consent mechanisms and approvals needed.

## Conclusions

Our engagement sessions raised very important issues, helped improve COPCOV key messages, re-assessed site-specific feasibility, reviewed certain safety aspects and facilitated embedding the views of the target population in the study. By conducting sessions at different timepoints and using a shared but flexible format it was possible to adapt to the changes in the pandemic and in our understanding of it and anticipate difficulties. Additionally, having a designated member of the study team responsible for engagement activities allowed for concerted efforts and for the uptake of relevant findings. Our experience strongly supports the use of participatory practices and the presence of dedicated engagement staff at research institutions and shows that even for large multi-centre trials and during a pandemic, organisational hurdles can be overcome. The fruits are well-worth the efforts.

## Data Availability

Zenodo: COPCOV engagement statements and questions. https://doi.org/10.5281/zenodo.5151543 ^36^. This project contains the following underlying data: -   COPCOV_engagement_questions.pdf (participant responses noted in the sessions) Data are available under the terms of the Creative Commons Attribution 4.0 International license (CC-BY 4.0).
