# Supplementary material for: Good participatory practice for coronavirus disease 2019 (COVID-19) research: the case of a COVID-19 prevention study
Source: Wellcome Open Res. 2022 Dec 1;6:216. Originally published 2021 Aug 24. [Version 3] doi: 10.12688/wellcomeopenres.16880.3 (PMC9971639; doi:10.12688/wellcomeopenres.16880.3)
Supplement: Peer review history

# Peer review: Reviewer response for version 3

**DOI:** 10.21956/wellcomeopenres.20526.r54484

**Published:** 2023-02-06

**Authors:** Anwesha Chakraborty

**Affiliations:** 1 Department of Political and Social Sciences, University of Bologna, Bologna, Italy

Overall, the paper has a sound research design and touches upon an important topic: participatory practices during clinical trials. The paper ends with the assertion that the use of participatory practices is a desirable goal. I have two main observations, both of which are related to the primary argument:

1. That participatory practices are important in any situation related to issues of public risks and safety is a well-established discussion in multiple academic discourses, especially, but not limited to, public understanding of science and technology; and clinical governance. I would like the authors to push this argument further and explain what is achieved through participation. Even if it is not one of themes that emerged from thematic analysis, it would still be a meaningful addition to the paper.
2. While the research design is inductive, the paper would benefit from a clear research question and/or research objective which needs to be mentioned in the first section of the paper.

Is the work clearly and accurately presented and does it cite the current literature?

Yes

If applicable, is the statistical analysis and its interpretation appropriate?

Not applicable

Are all the source data underlying the results available to ensure full reproducibility?

Yes

Is the study design appropriate and is the work technically sound?

Yes

Are the conclusions drawn adequately supported by the results?

Yes

Are sufficient details of methods and analysis provided to allow replication by others?

Yes

Reviewer Expertise:

Public understanding of science; responsible research and innovation; participatory governance

I confirm that I have read this submission and believe that I have an appropriate level of expertise to confirm that it is of an acceptable scientific standard.

# Response

**Published:** 2023-04-04

**Authors:** Carlo Perrone

**Affiliations:** Mahidol Oxford Tropical Medicine Research Unit, Thailand

Dear Dr Chakraborty,  

 Thank you very much for your review and insight.  

 On the first point- I completely agree. Through participation, public understanding of science and the quality of the scientific process and research will increase. This will benefit both researchers and the community they are trying to serve. Through participation, communities can play an active role in planning research or at least better understand the research that is being carried out in their communities.  

 On the second point – This paper describes a series of engagement activities rather than a research study with clearly defined research questions. When designing our engagement activities, we decided to keep the discussion as broad as possible so to obtain feedback on a wide range of topics including issues we may not have thought of at the planning stage. For this reason, a concrete, specific question was not formulated in advance. An approach such as ours has of course downsides, so that questions facilitators or the engagement coordinators may like to have feedback on could be neglected by participants. When planning the activities, advantages, and disadvantages of a broad versus focused approach must be thoroughly weighed.  

 Best regards, Carlo Perrone

# Peer review: Reviewer response for version 3

**DOI:** 10.21956/wellcomeopenres.20526.r53529

**Published:** 2023-01-18

**Authors:** Shelley Lees

**Affiliations:** 1 Department of Global Health and Development, London School of Hygiene & Tropical Medicine, London, UK

This is now a much improved and interesting paper.

Is the work clearly and accurately presented and does it cite the current literature?

No

If applicable, is the statistical analysis and its interpretation appropriate?

Not applicable

Are all the source data underlying the results available to ensure full reproducibility?

No

Is the study design appropriate and is the work technically sound?

No

Are the conclusions drawn adequately supported by the results?

No

Are sufficient details of methods and analysis provided to allow replication by others?

No

Reviewer Expertise:

Anthropology of epidemics and clinical trials

I confirm that I have read this submission and believe that I have an appropriate level of expertise to confirm that it is of an acceptable scientific standard.

# Peer review: Reviewer response for version 2

**DOI:** 10.21956/wellcomeopenres.19263.r51635

**Published:** 2022-08-15

**Authors:** Cathrine Slack

**Affiliations:** 1 HIV AIDS Vaccines Ethics Group, University of KwaZulu-Natal, Pietermaritzburg, South Africa

Stakeholder engagement is an important component of research, as captured in leading international ethics guidelines. Efforts to share engagement approaches and critically reflect on these are important for the field. There are several ways this paper could be further strengthened, as set out below:

**Abstract:**

- The authors should locate the study in current ethics guidelines.
- They should describe the aims before describing the method.
- The stated aims are “ *to assess the feasibility of the study at the respective sites, to identify context-specific ethical issues, to understand concerns potential participants might have, to fine tune research procedures and to refine COPCOV information materials*” however the methods are stated quite narrowly and could resonate better with the more broadly stated aims.
- Under the results, the stated results only address willingness, and concerns - which is a smaller subset of the stated aims.

**Methods/ approvals and ethical considerations:**

- CIOMS (2016) recommends that researchers and RECs should be cognizant of the point “ *at which the process of community engagement becomes a stage of formative research that itself requires ethics review. Examples of community engagement processes that may require ethics review include systematic data collection that can be generalized and disseminated in forums outside of the community* *(…)*”(p.26). The engagement being described in this paper might have some key lessons for other researchers on the particular point raised in CIOMS (2016). The authors should reflect on whether the initial purpose of canvassing stakeholders was to plan for better engagement/research or whether the initial purpose of canvassing stakeholders was to develop generalizable knowledge for publication; or indeed whether the purpose(s) of canvassing stakeholders evolved over time or in response to inputs. The fact that CIOMS (2016) contains guidance on this issue (i.e. when those whose views are being canvassed are being involved in an *engagement activity* versus when those being engaged are being enrolled in a *study*) suggests that it is a tricky concern that researchers should remain mindful of and think carefully about. The authors should consider what they have learned on this issue, and make recommendations to help others.

**Methods/ Procedure:**

- The text states “ *Participants of the sessions were briefed on the COPCOV study design and procedures (10–20 minutes) and asked two predefined questions in the local language: 1) “Would you take part in such a study and why?” and 2) “What additional information would strengthen or make you change your decision?*” Describe what questions were posed to address the other aims.

**Results:**

- Describe whether the attendees gave permission to be quoted even in an anonymized way.

**Strengths and limitations**

- This section states “ *This was primarily due to the feasibility and practical reasons such as availability of experienced engagement staff at the respective sites*” Comment on the recommendation in ethics guidelines that sites have funded and staffed engagement initiatives (e.g. GPP HIV 2011; GPP-EP 2016).
- “ *Due to the timing of the engagement sessions, there was a risk that participants felt their concerns were unanswered*”. Describe the feedback that was given to attendees, or the sites from which they were drawn, on the findings from this project. This would be respectful of their contributions.

**References:**

- The authors should refer to the updated 2016 version of CIOMS.

Thank you for the opportunity to learn about this engagement activity and to comment on the manuscript.

Is the work clearly and accurately presented and does it cite the current literature?

Yes

If applicable, is the statistical analysis and its interpretation appropriate?

Not applicable

Are all the source data underlying the results available to ensure full reproducibility?

No source data required

Is the study design appropriate and is the work technically sound?

Yes

Are the conclusions drawn adequately supported by the results?

Yes

Are sufficient details of methods and analysis provided to allow replication by others?

Yes

Reviewer Expertise:

Research ethics, ethics of clinical trials, stakeholder engagement

I confirm that I have read this submission and believe that I have an appropriate level of expertise to confirm that it is of an acceptable scientific standard, however I have significant reservations, as outlined above.

# Response

**Published:** 2022-10-24

**Authors:** Carlo Perrone

**Affiliations:** Mahidol Oxford Tropical Medicine Research Unit, Thailand

Dear Dr. Slack,

 On behalf of the authors I wish to thank you for your review, you raised some very relevant points that we answer as follows: 

 Abstract:

- The authors should locate the study in current ethics guidelines. Sentence added in abstract (Methods section)
- They should describe the aims before describing the method. Switched, aims now in background.
- The stated aims are “ *to assess the feasibility of the study at the respective sites, to identify context-specific ethical issues, to understand concerns potential participants might have, to fine tune research procedures and to refine COPCOV information materials*” however the methods are stated quite narrowly and could resonate better with the more broadly stated aims. --> **the questions were broad and centred on reasons for willingness or unwillingness to participate, as well as open questions in general. As we aimed to answer the aims through the questions above, no tailored questions for the other aims were asked.**

Under the results, the stated results only address willingness, and concerns - which is a smaller subset of the stated aims.  - **> Made clearer in abstract, grouped results and conclusions together.**

 Methods/ approvals and ethical considerations:

- CIOMS (2016) recommends that researchers and RECs should be cognizant of the point “ *at which the process of community engagement becomes a stage of formative research that itself requires ethics review. Examples of community engagement processes that may require ethics review include systematic data collection that can be generalized and disseminated in forums outside of the community* *(…)*”(p.26). The engagement being described in this paper might have some key lessons for other researchers on the particular point raised in CIOMS (2016). The authors should reflect on whether the initial purpose of canvassing stakeholders was to plan for better engagement/research or whether the initial purpose of canvassing stakeholders was to develop generalizable knowledge for publication; or indeed whether the purpose(s) of canvassing stakeholders evolved over time or in response to inputs. The fact that CIOMS (2016) contains guidance on this issue (i.e. when those whose views are being canvassed are being involved in an *engagement activity* versus when those being engaged are being enrolled in a *study*) suggests that it is a tricky concern that researchers should remain mindful of and think carefully about. The authors should consider what they have learned on this issue, and make recommendations to help others. **-> We added “lessons learnt” to “strengths and limitations” and added a new paragraph at the end.**

Methods/ Procedure:

- The text states “ *Participants of the sessions were briefed on the COPCOV study design and procedures (10–20 minutes) and asked two predefined questions in the local language: 1) “Would you take part in such a study and why?” and 2) “What additional information would strengthen or make you change your decision?*” Describe what questions were posed to address the other aims.- **> No additional questions were posed, see answer above.**

Results:

- Describe whether the attendees gave permission to be quoted even in an anonymized way. We did not ask specific permission for participants to be quoted. -> **Our protocol which included the engagement activities was approved relevant ethics committees. The ethics committees which approved our protocol (including the engagement work) did not require us to get specific written consent for engagement or use of anonymised quotes. This is described under ‘Approvals and Ethical Considerations’.**
**We only used quotes that were completely anonymised and could not be traced back to individuals. Quotes that were long and could potentially identify individuals through idiolect or had references to their specific job or place of work were not included. The topics discussed were not sensitive (e.g. related to their own health or revealing confidential information) but rather their opinions on a research project of which details were publicly available e.g. on our institutional and funder websites. **​​​​​​​

Strengths and limitations

- This section states “ *This was primarily due to the feasibility and practical reasons such as availability of experienced engagement staff at the respective sites*” Comment on the recommendation in ethics guidelines that sites have funded and staffed engagement initiatives (e.g. GPP HIV 2011; GPP-EP 2016). **-> Good point, added a comment in “strengths, limitations and lessons learnt” paragraph and in “Conclusions”.**
- “ *Due to the timing of the engagement sessions, there was a risk that participants felt their concerns were unanswered*”. Describe the feedback that was given to attendees, or the sites from which they were drawn, on the findings from this project. This would be respectful of their contributions. -> **We agree. We will provide a lay report to all participating sites that includes the results of the COPCOV study and engagement activities after the completion of the COPCOV study**.

# Peer review: Reviewer response for version 2

**DOI:** 10.21956/wellcomeopenres.19263.r47219

**Published:** 2022-01-18

**Authors:** Shelley Lees

**Affiliations:** 1 Department of Global Health and Development, London School of Hygiene & Tropical Medicine, London, UK

Thank you to authors who have addressed all of my comments on the paper. The paper has now addressed the wider debates about GPP in the introduction and made it clearer that this is a research study. The authors have also expanded the strengths and limitations. The paper now stands as a contribution to the literature on the important role of GPP in clinical trials during a pandemic

Is the work clearly and accurately presented and does it cite the current literature?

No

If applicable, is the statistical analysis and its interpretation appropriate?

Not applicable

Are all the source data underlying the results available to ensure full reproducibility?

No

Is the study design appropriate and is the work technically sound?

No

Are the conclusions drawn adequately supported by the results?

No

Are sufficient details of methods and analysis provided to allow replication by others?

No

Reviewer Expertise:

Anthropology of epidemics and clinical trials

I confirm that I have read this submission and believe that I have an appropriate level of expertise to confirm that it is of an acceptable scientific standard.

# Peer review: Reviewer response for version 1

**DOI:** 10.21956/wellcomeopenres.18621.r45632

**Published:** 2021-09-13

**Authors:** Shelley Lees

**Affiliations:** 1 Department of Global Health and Development, London School of Hygiene & Tropical Medicine, London, UK

This paper has some potential but as it stands it falls short of reporting good participatory practice. Firstly, the introduction is very short and does not delve into the debates around GPP. There is much literature on the subject, presenting different methods of GPP for clinical trials. The methods section describes the engagement sessions but does not suggest this is a research study. Whilst the attendees were given information that the findings from the sessions would be included in reports, they did not give informed consent for this to be a data collection exercise, as far as I can see.

 The findings are about the data from the sessions as if this was a data collection exercise. I had expected to see the findings reporting on how the engagement exercise impacted on trial procedures as well as communication about the trial.

 The findings, rather than draw on the debates about GPP, were mainly conjecture and included some findings not reported in the findings section. For example, the sentence " ..the participants thought that the trial would challenge participants by infecting them with the virus".

 There is some discussion about the limits of a single engagement exercise on influencing trial design, but I think this gets lost in the summary of the findings. This section should be expanded to discuss how better engagement could be set up at design stage of trials.

 Overall, I think the paper should acknowledge that the participants were educated in medicines as most were health care workers. This means that their concerns about testing drugs were based on good knowledge. I think the authors would better use this to exemplify the limits of such engagement approaches and rather discuss more effective engagement taking into account the difficulties of engaging rapidly during a pandemic.

Is the work clearly and accurately presented and does it cite the current literature?

No

If applicable, is the statistical analysis and its interpretation appropriate?

Not applicable

Are all the source data underlying the results available to ensure full reproducibility?

No

Is the study design appropriate and is the work technically sound?

No

Are the conclusions drawn adequately supported by the results?

No

Are sufficient details of methods and analysis provided to allow replication by others?

No

Reviewer Expertise:

Anthropology of epidemics and clinical trials

I confirm that I have read this submission and believe that I have an appropriate level of expertise to state that I do not consider it to be of an acceptable scientific standard, for reasons outlined above.

# Response

**Published:** 2021-11-17

**Authors:** Carlo Perrone

**Affiliations:** Mahidol Oxford Tropical Medicine Research Unit, Thailand

We thank the reviewer for her valued comments. Here is a point by point response to reviewer.

 Comment: This paper has some potential but as it stands it falls short of reporting good participatory practice. Firstly, the introduction is very short and does not delve into the debates around GPP. There is much literature on the subject, presenting different methods of GPP for clinical trials.

 Response: We have now included a section discussing the literature in GPP and added relevant references.

 Comment: The methods section describes the engagement sessions but does not suggest this is a research study. Whilst the attendees were given information that the findings from the sessions would be included in reports, they did not give informed consent for this to be a data collection exercise, as far as I can see.

 Response: We are reporting the outcomes of engagement rather than research. We have added a few sentence in the Methods (Approvals and ethical considerations) to make it clearer. All participants provided verbal consent. We have also added a sentence to explain this.

 Comment: The findings are about the data from the sessions as if this was a data collection exercise. I had expected to see the findings reporting on how the engagement exercise impacted on trial procedures as well as communication about the trial.

 Response: We have added a few sentences in the Discussion section (Implications on the COPCOV study). Additionally we have provided specific examples of how this engagement exercise impacted our trial.

 Comment: The findings, rather than draw on the debates about GPP, were mainly conjecture and included some findings not reported in the findings section. For example, the sentence " ..the participants thought that the trial would challenge participants by infecting them with the virus".

 Response: This was an error. We have now included this in the Results section “Concerns about safety and risk-benefit balance”

 Comment: There is some discussion about the limits of a single engagement exercise on influencing trial design, but I think this gets lost in the summary of the findings. This section should be expanded to discuss how better engagement could be set up at design stage of trials.

 Response: We have now expanded this section.

 Comment: Overall, I think the paper should acknowledge that the participants were educated in medicines as most were health care workers. This means that their concerns about testing drugs were based on good knowledge. I think the authors would better use this to exemplify the limits of such engagement approaches and rather discuss more effective engagement taking into account the difficulties of engaging rapidly during a pandemic.

 Response: We have added a sentence in the Limitation section
